# Enzymatic sugar production from elephant grass and reed straw through pretreatments and hydrolysis with addition of thioredoxin-His-S

**DOI:** 10.1186/s13068-019-1629-y

**Published:** 2019-12-27

**Authors:** Xianqin Lu, Can Li, Shengkui Zhang, Xiaohan Wang, Wenqing Zhang, Shouguo Wang, Tao Xia

**Affiliations:** 1 State Key Laboratory of Biobased Material and Green Papermaking, Qilu University of Technology, Jinan, 250353 Shandong People’s Republic of China; 2 School of Bioengineering, Qilu University of Technology, Jinan, 250353 Shandong People’s Republic of China; 3 Advanced Research Institute for Multidisciplinary Science, Qilu University of Technology, Jinan, 250353 Shandong People’s Republic of China

**Keywords:** Elephant grass, Reed straw, Pretreatment, Enzymatic hydrolysis, Thioredoxin-His-S

## Abstract

**Background:**

The bioconversion of lignocellulose to fermentable C5/C6-saccharides is composed of pretreatment and enzymatic hydrolysis. Lignin, as one of the main components, resists lignocellulose to be bio-digested. Alkali and organosolv treatments were reported to be able to delignify feedstocks and loose lignocellulose structure. In addition, the use of additives was an alternative way to block lignin and reduce the binding of cellulases to lignin during hydrolysis. However, the relatively high cost of these additives limits their commercial application.

**Results:**

This study explored the feasibility of using elephant grass (*Pennisetum purpureum*) and reed straw (*Phragmites australis*), both of which are important fibrous plants with high biomass, no-occupation of cultivated land, and soil phytoremediation, as feedstocks for bio-saccharification. Compared with typical agricultural residues, elephant grass and reed straw contained high contents of cellulose and hemicellulose. However, lignin droplets on the surface of elephant grass and the high lignin content in reed straw limited their hydrolysis performances. High hydrolysis yield was obtained for reed straw after organosolv and alkali pretreatments via increasing cellulose content and removing lignin. However, the hydrolysis of elephant grass was only enhanced by organosolv pretreatment. Further study showed that the addition of bovine serum albumin (BSA) or thioredoxin with His- and S-Tags (Trx-His-S) improved the hydrolysis of alkali-pretreated elephant grass. In particular, Trx-His-S was first used as an additive in lignocellulose saccharification. Its structural and catalytic properties were supposed to be beneficial for enzymatic hydrolysis.

**Conclusions:**

Elephant grass and reed straw could be used as feedstocks for bioconversion. Organosolv and alkali pretreatments improved their enzymatic sugar production; however, the increase in hydrolysis yield of pretreated elephant grass was not as effective as that of reed straw. During the hydrolysis of alkali-pretreated elephant grass, Trx-His-S performed well as additive, and its structural and catalytic capability was beneficial for enzymatic hydrolysis.

## Background

Lignocellulose is the most abundant renewable macromolecule available on the earth and can be bioconverted to biofuels or chemicals, which is green, sustainable, and environment friendly [1, 2]. Cellulose and hemicellulose, the main components in lignocellulose, can be bio-digested to C5/C6-saccharides and can furthermore be converted to furfural and other value-added chemicals [3, 4]. Lignin, one of the main components in lignocellulose, cross-links with hemicellulose and cellulose, thus resulting in the resistance of lignocellulose to bio-digestion [5]. As an essential step during the bioconversion of lignocellulose, pretreatment is performed before enzymatic hydrolysis, with the aim to decrease recalcitrance and enhance enzymatic digestibility [5, 6].

Many pretreatment methods have been developed with various solvents or catalysts, such as alkali, acid, organic solvents, and ionic liquid [4, 7]. Among these methods, alkali pretreatment was able to exert strong delignification capability by breaking the cross-linkage between lignin and hemicellulose and by increasing the porosity of the substrate [8]. Sun et al. suggested that the pretreatment with the diluted alkali could swell lignocellulosic materials, which led to a decrease in the degrees of polymerization and crystallinity [9]. Organosolv pretreatment was also reported as an alternative method to delignify and modify the structure of lignin, which could also break the fibrous structure and reduce the degree of crystallinity [7]. Delignification and a loose structure were in favor to increase the access of cellulase to cellulose, which resulted in high sugar yield.

Enzymatic hydrolysis, the essential step to depolymerization of lignocellulose to reducing sugar, was performed following pretreatment. Currently, the cost of the used enzymes hindered the commercial application of bioconversion. Pretreatment was one way to reduce the dosage of enzyme. Meanwhile, the use of additives was an alternative to block lignin, reduce the adsorption of cellulases to lignin, and increase enzymatic efficiency during hydrolysis [10–13]. Although BSA and Tween series were effective additives, their high cost limited their commercial application [11, 13]. It has been reported that carbohydrate-binding modules (CBMs) of cellulases and fibronectin III-like domains (FnIIIs) of beta-glucosidase played an important role in the adsorption of cellulase to lignin [14, 15]. Our previous research suggested that thioredoxin with His-Tag and S-Tag (Trx-His-S) showed high binding affinity to lignin than CBMs and FnIIIs. In addition, Trx-His-S could be easily expressed in *Escherichia coli* and achieved a high expression level. Due to these results, the business adaptive Trx-His-S could be used as a hydrolysis additive to competitively bind lignin with cellulase and thus increase the enzymatic hydrolysis efficiency.

The origin of raw material also played an important role in the bioconversion process. The potential use of agricultural residues and energy grass as substrates for producing bio-ethanol has attracted increasing attention, due to its high yield and non-competition with fertile lands, forests, and food crops [16]. Recently, research on bioconversion mainly focused on corn stover and wheat straw. Meanwhile, a number of low-cost feedstocks were elevated [3]. This study selected elephant grass and reed straw as the feedstocks to study their feasibility for bioconversion. Elephant grass achieves large biomass production of about 45 t/ha and can be harvested 3–4 times per year. Moreover, elephant grass can grow and survive in many soil and weather conditions. Previous research showed that elephant grass, which was similar to sugarcane, was an alternative feedstock for bioconversion [17, 18]. Reed straw is a traditional pulping material with high cellulose content and good fiber properties, which is an abundant wetland lignocellulose plant with wide distribution throughout Asia and Europe [19, 20]. In China, the planting area of reed straw exceeded 0.67 million hectares, and the annual production exceeds 3 million tons [19]. Reed straw can also be used for the phytoremediation of wastewater, soil, and sediments [20]. As described above, elephant grass and reed straw as feedstocks were not only able to provide abundant materials for bioconversion, but also achieved environmental benefits.

Although elephant grass and reed straw have great potential for application in bioconversion, present researches on their lignocellulose properties in different pretreatments and hydrolysis conditions are relatively limited. This study analyzed the structural properties of elephant grass and reed straw, such as the compositions, surficial properties, and distribution of lignin, in comparison with the typical agricultural residues, corn stover, and wheat straw. Subsequently, organosolv and alkali treatments were conducted to break the recalcitrance to hydrolysis. The hydrolysis of elephant grass was optimized by addition of BSA and Trx-His-S, in which Trx-His-S was first used as an additive during lignocellulose bioconversion, which achieved good performance. The possible role of Trx-His-S in hydrolysis was discussed by studying the structural properties and the catalytic capability of Trx-His-S.

## Results and discussion

### Chemical compositions and bioconversion capability of reed straw and elephant grass

Table 1 shows the chemical compositions of corn stover, wheat straw, reed straw, and elephant grass. The cellulose contents of elephant grass (40.30%) and reed straw (38.52%) were higher than those of wheat straw (35.37%) and corn stover (32.56%). However, the lignin contents of reed straw (26.62%) and elephant grass (21.68%) were slightly higher than those of wheat straw (21.53%) and corn stover (17.14%). In addition, a relatively higher hemicellulose content of reed straw (18.85%) was also observed, in which 13.97%, 15.51%, and 9.19% of hemicellulose was obtained for corn stover, wheat straw, and elephant grass, respectively.

**Table 1:** Chemical compositions of corn stover, wheat straw, reed straw, and elephant grass (%)

	Cellulose	Lignin			Hemicellulose
		Soluble acid	Insoluble acid	Total	
Corn stover	32.56	1.66	15.49	17.14	13.97
Wheat straw	35.37	1.38	20.15	21.53	15.51
Reed straw	38.52	1.03	25.59	26.62	18.85
Elephant grass	40.30	0.80	20.87	21.68	9.19

**Fig. 1:**
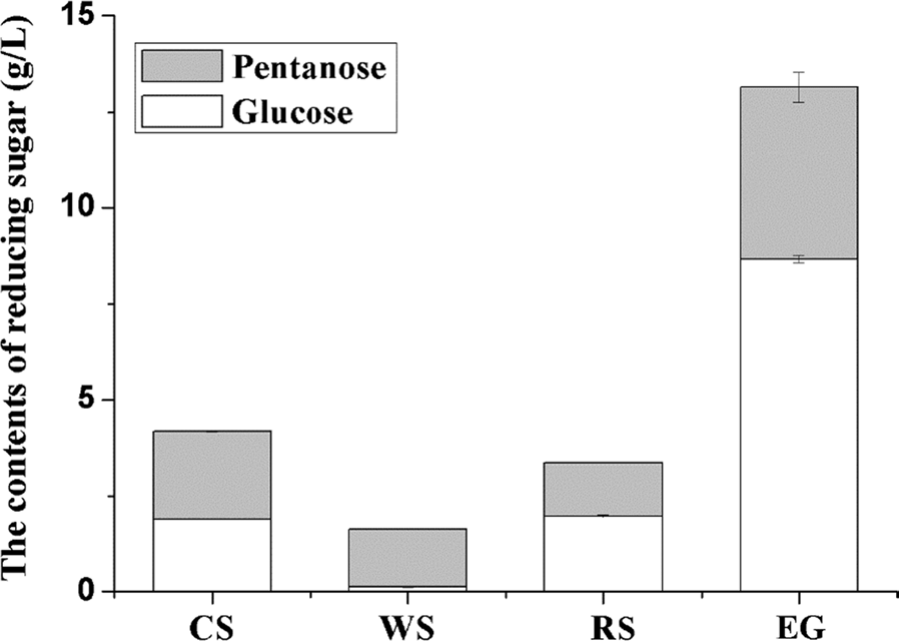
Contents of pentose and glucose produced by hydrolysis of corn stover (CS), wheat straw (WS), reed straw (RS), and elephant grass (EG)

After the hydrolysis of corn stover (CS), wheat straw (WS), reed straw (RS), and elephant grass (EG), the content of produced reducing sugar was determined (Fig. 1). The results showed that the generated sugar for elephant grass was higher than others, reaching 13.14 g/L. Although the holocellulose (cellulose and hemicellulose) content of reed straw was relatively higher, the hydrolysis of reed straw acquired a lower sugar yield than that of corn stover.

Cellulose, hemicellulose, and lignin were the main components of biomass. Cellulose and hemicellulose could be hydrolyzed to fermentable sugar and further be fermented to biofuels or other chemicals. However, the lignin cross-linked with cellulose and hemicellulose, thus restricting lignocellulose from being digested. Meanwhile, the lignin could competitively adsorb cellulase with cellulose and reduce the hydrolysis process during bio-hydrolysis [5]. Thus, the high contents of lignin and hemicellulose may cause the stronger restriction of reed straw to bio-hydrolysis, leading to the poor hydrolysis production of reed straw, whereas the high cellulose content may lead to the high enzymatic hydrolysis efficiency of elephant grass.

### Morphological properties

**Fig. 2:**
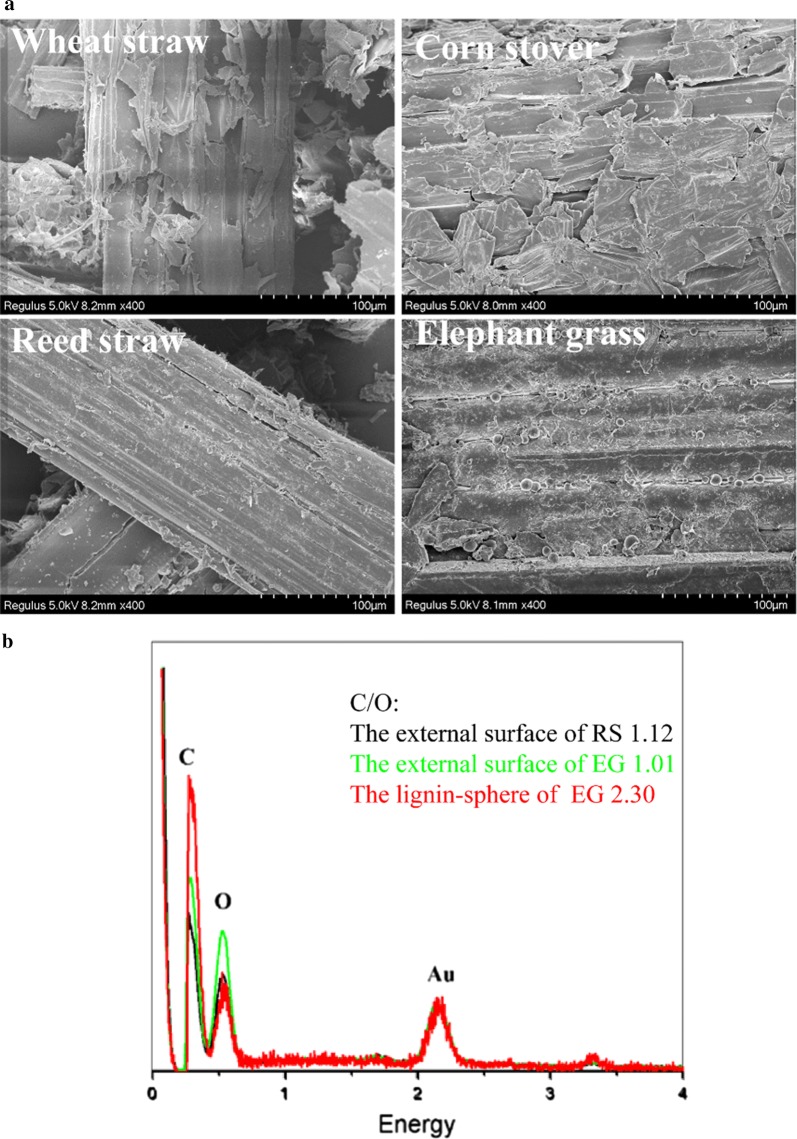
Morphological properties of feedstocks. **a** The external surface of wheat straw, corn stover, reed straw, and elephant grass by SEM imaging. **b** The EDS mapping of carbon, oxygen, and Au ion in the external surface of reed straw (RS), elephant grass (EG), and the spheres on the elephant grass surface

Figure 2 shows the external surfaces’ structures of wheat straw, elephant grass, reed straw, and corn stover by SEM imaging. In the images of wheat straw and corn stover, many loosened fiber fragments were distributed on the external surface. The surfaces of reed straw and elephant grass were relatively tight and smooth, compared with wheat straw and corn stover. As described above, the relatively high contents of lignin and hemicellulose in reed straw and elephant grass may induce the tight fiber structure, especially for reed straw [21]. The tight external surface structure reduced the cellulose accessibility to enzymes and significantly affected the efficiency of bio-hydrolysis.

Figure 2a shows that many spheres were distributed on the external surface of elephant grass. The spherical droplets were used to be observed on the surface of hardwood pretreated by liquid hot water, which was identified as lignin [22]. A previous report indicated that the formation of lignin droplets on cell walls typically occurs during hydrothermal pretreatment [23]. For elephant grass, the growing environment, hot and humid tropical climates, possibly induced the formation of lignin sphere, which was similar to hydrothermal pretreatment.

Figure 2b shows the energy dispersion spectrum (EDS) mapping of carbon and oxygen on the external surface of reed straw and elephant grass and the lignin droplets on the elephant grass. The C/O ratio in lignin is significantly higher than that in cellulose. Thus, higher C/O ratio indicated more lignin content [24]. As shown in Fig. 2b, the C/O ratio of reed straw external surface (1.12) was higher than that of elephant grass external surface (1.01), which agreed with the high content of lignin in reed and the tight surface structure as described above. For the surface of lignin sphere, the C/O ratio (2.30) was significantly higher than that of elephant grass (1.01), which further confirmed the high lignin component in spheres. The distribution of lignin droplets hindered enzymatic hydrolysis efficiency of elephant grass, by deterring cellulase to access cellulose and nonproductively adsorbing to cellulase [23].

The X-ray diffraction (XRD) patterns were measured to analyze the crystalline index of the feedstock. The crystallinity was calculated based on the characteristic absorption peaks of crystalline cellulose at 16° and 23°. The crystalline indexes were 50.22, 46.22, 49.45, and 54.81 for corn stover, wheat straw, reed straw, and elephant grass, respectively. The higher crystalline index indicated stronger interactions between cellulose sheets, which resulted in weaker hydrophilicity, stronger stability, and recalcitrance to biodegradation [25]. These results demonstrated that elephant grass obtained high content of cellulose, but also acquired strong crystallinity. Thus, although the hydrolysis of elephant grass produced the highest yield of reducing sugar among four feedstocks, breaking cellulose crystallinity and dissolving surface lignin spheres may further promote the production process of reducing sugar.

### Organosolv and alkali pretreatments and hydrolysis

**Fig. 3:**
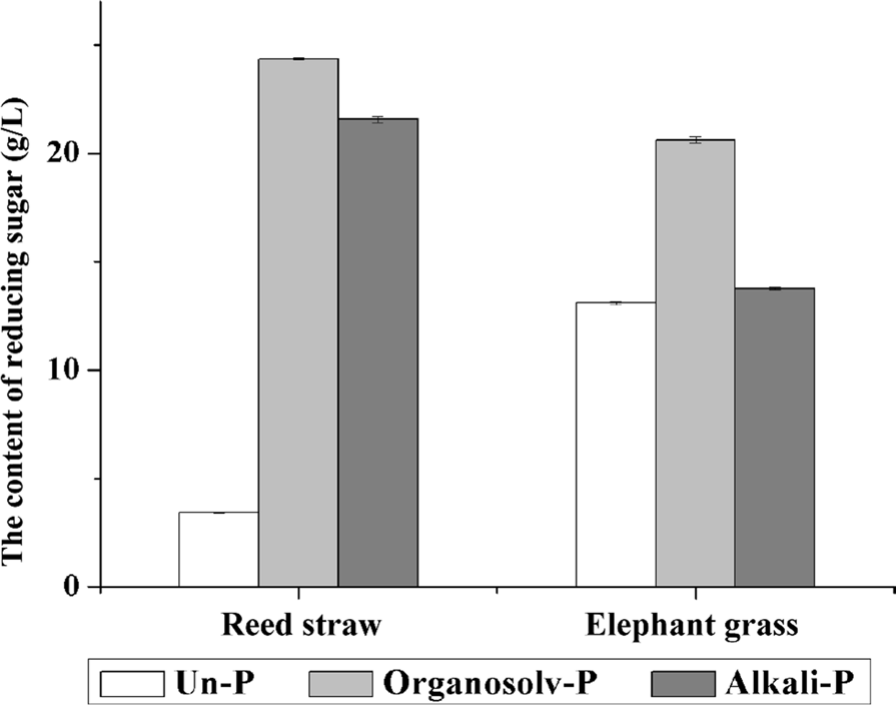
Content of reducing sugar produced by hydrolysis of reed straw and elephant grass without pretreatment (Un-P) and with organosolv pretreatment (Organosolv-P) and alkali pretreatment (Alkali-P)

Since organosolv and alkali treatments have been reported to be able to deconstruct and solubilize lignin and decrease the degree of polymerization and crystallinity [7], these pretreatments were performed in this study. As shown in Fig. 3, the reducing sugar yield of hydrolysis of reed straw and elephant grass clearly increased after organosolv and alkali pretreatments, especially that of reed straw. During the hydrolysis of reed straw, the produced reducing sugar ranged from 3.46 to 24.34 g/L after organosolv pretreatment and was to 21.67 g/L after alkali pretreatment. The reducing sugar produced by the hydrolysis of elephant grass ranged from 13.14 to 20.73 g/L after organosolv pretreatment, while the reducing sugar yield only slightly increased after alkali pretreatment.

Table 2 presents the chemistry fractions of cellulose, lignin, and hemicellulose after both pretreatments. In reed straw, the cellulose fraction increased from 38.52 to 69.46% and to 55.88% after organosolv and alkali pretreatments, respectively, while the lignin and hemicellulose in organosolv- and alkali-pretreated reed straw reduced remarkably. In particular, in organosolv-pretreated reed straw, the lignin was reduced from 26.62 to 13.10% and hemicellulose was reduced from 18.85 to 8.71%. Alkali pretreatment was reported to have a strong delignification capability, which was able to break the cross-links between lignin and hemicellulose [8]. And the organosolv pretreatment was an alternative way to modify and dissolve lignin [7]. Thus, the dissolution of lignin during pretreatments led to the relatively high cellulose content in the feedstock.

**Table 2:** Chemical compositions of reed straw (RS) and elephant grass (EG) with and without pretreatments (%)

	Cellulose	Lignin			Hemicellulose
		Soluble acid	Insoluble acid	Total	
RS					
Un-P	38.52	1.03	25.59	26.62	18.85
Organosolv-P	69.46	0.64	12.46	13.10	8.71
Alkali-P	55.88	0.82	18.57	19.39	17.45
EG					
Un-P	40.30	0.80	20.87	21.68	9.19
Organosolv-P	72.01	0.68	13.52	14.20	8.65
Alkali-P	60.12	0.81	17.15	17.96	15.76

**Fig. 4:**
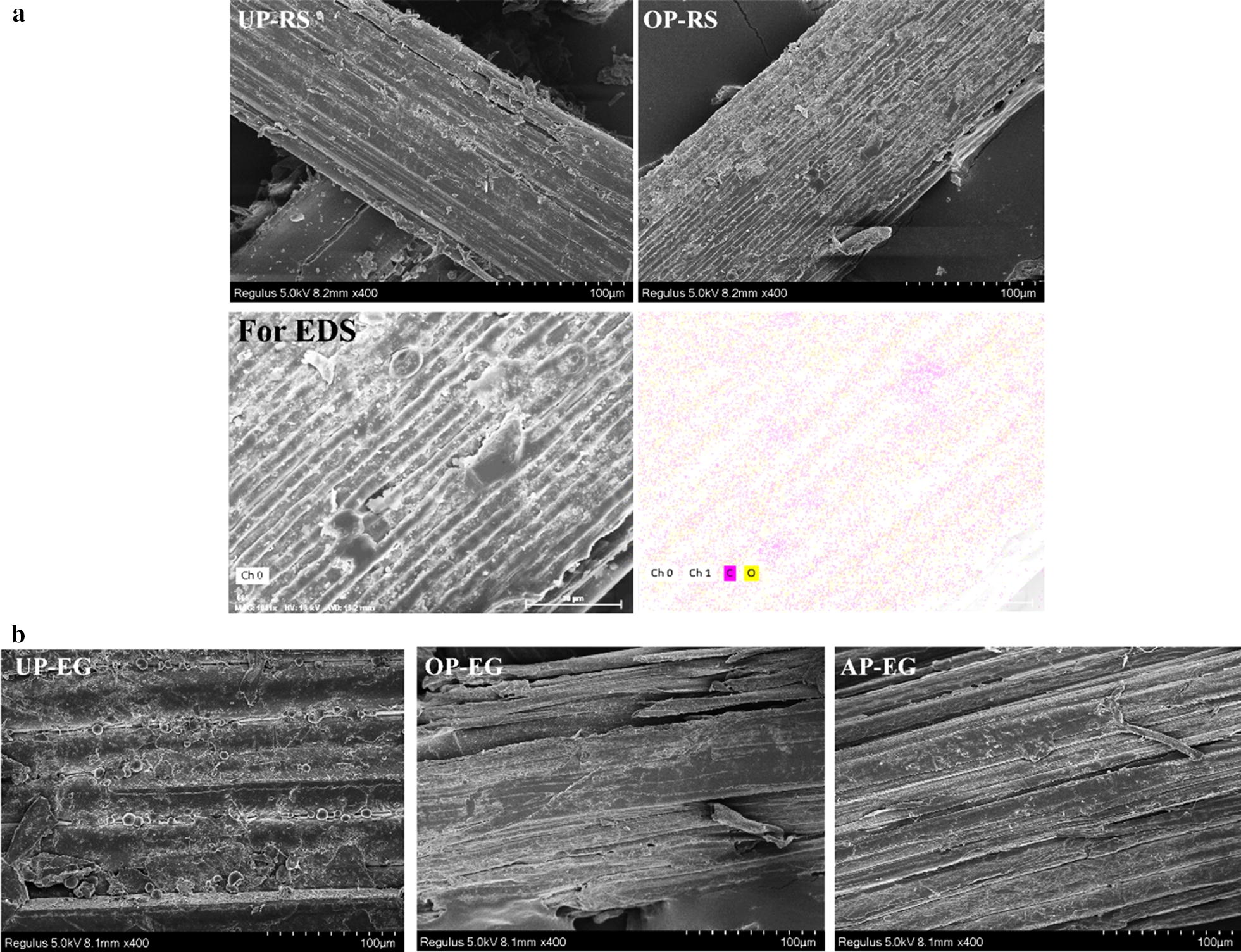
Morphological properties of reed straw and elephant grass by SEM imaging. **a** The external surface of reed straw (RS) without pretreatment (UP-RS) and with organosolv pretreatment (OP). And the EDS mapping of carbon and oxygen in reed straw after organosolv pretreatment. **b** The external surface of elephant grass (EG) without pretreatment (UP-EG) and with organosolv pretreatment (OP-EG) and alkali pretreatment (AP-EG)

The results of SEM imaging (Fig. 4) confirmed the removal of lignin after pretreatment. The external protective structures on reed straw surface with high C/O ratio were dissolved, and the fibers were exposed. This may lead to the high hydrolysis yield of reed straw after organosolv and alkali pretreatments. Many studies suggested that the dissolution of lignin and hemicellulose favored bio-digestion and the conversion of lignocellulose to fermentable sugars by relieving the physical obstruction to hydrolysis and increasing the accession of cellulose to cellulase [5, 6, 26, 27]. These data also supported our above opinion.

In elephant grass, the cellulose fraction increased from 40.30 to 72.01% and 60.01% after organosolv and alkali pretreatments, respectively. After organosolv pretreatment, the lignin content of elephant grass decreased from 21.68 to 14.20% and the hemicellulose content decreased from 9.19 to 8.65%. SEM imaging (Fig. 4) also suggested that the lignin sphere on the surface was dissolved during pretreatment, and the fibers were swollen. After alkali pretreatment, the hemicellulose content of elephant grass increased unexpectedly from 9.19 to 15.76% and lignin was only reduced from 21.68 to 17.96%. As described above, in elephant grass, alkali pretreatment was not as effective as organosolv pretreatment on lignin deconstruction and dissolution of hemicellulose, which maybe the reason for the slight enhancement of enzymatic digestibility after alkali pretreatment.

### Saccharification of elephant grass with different additives

**Fig. 5:**
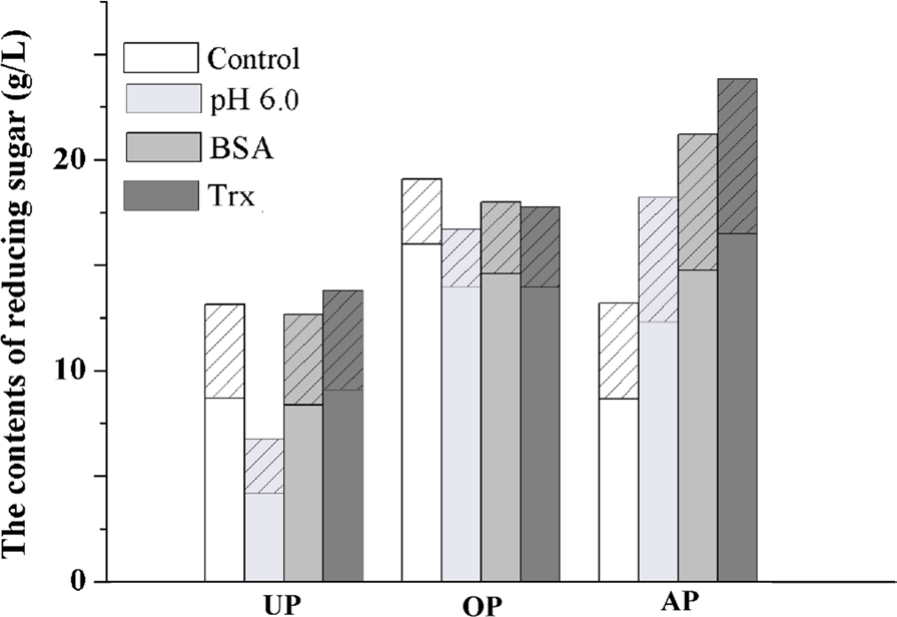
Content of reducing sugar produced by hydrolysis of elephant grass. The unpretreated (UP), organosolv-pretreated (OP), and alkali-pretreated (AP) elephant grass were hydrolyzed with higher pH value 6.0 (pH 6.0), or addition of BSA (BSA) and Trx-His-S (Trx). The reducing sugar included glucoses (the blank part) and pentanose (the slash part)

Pretreatment improved the hydrolysis efficiency, but the increase in hydrolysis yield of pretreated elephant grass was not as effective as that of reed straw. To address this issue, the saccharification process of elephant grass was optimized [11]. Figure 5 shows the yield of reducing sugar produced after hydrolysis of elephant grass without pretreatment (EG-UP), organosolv (EG-OP), and alkali (EG-AP) pretreatment tests by elevating the hydrolysis pH value and adding BSA or Trx-His-S.

According to our previous report, elevating the pH value can reduce the adsorption of cellulase to lignin by changing the protein surface properties and by enhancing the repulsion between lignin and enzyme, thus leading to an improvement in the hydrolysis yield [28]. As shown in Fig. 5, increasing the pH value, from pH 4.8 to pH 6.0, significantly increased the hydrolysis yield of EG-AP, while the yield of reducing sugar decreased in EG-UP and EG-OP. Because the pH 6.0 was not the optimal pH value for cellulase catalytic behavior, it may lead to a lower yield of reducing sugar in EG-UP and EG-OP. EG-AP had relatively high lignin content, in which the nonproductive adsorption of cellulase to lignin likely played a significant role in the reduction of the access of cellulase to cellulose and in hindering the hydrolytic process. Thus, increasing the pH value and relaxing the effect of lignin improved the hydrolysis yield of EG-AP.

BSA, as a non-enzymatic protein additive, showed high binding capability to lignin during enzymatic hydrolysis [13]. Addition of BSA during enzymatic hydrolysis could reduce the un-productive binding of cellulase to lignin and thus increase the hydrolysis efficiency [29]. The improvement in the hydrolysis yield of EG-AP confirmed the good performance of BSA, as shown in Fig. 5.

Trx-His-S was also added in the hydrolysis process. Notably, Trx-His-S exerted a better effect than BSA on the conversion of EG-AP, which increased the conversion production by 80%. With the addition of Trx-His-S, EG-AP produced more reducing sugar than organosolv-pretreated EG-AP, in which the produced glucose of EG-AP was similar to that of EG-OP, but the hexose produced was clearly higher than the latter. This was the first study on the usage of Trx-His-S as an additive in the bioconversion of lignocelluloses.

**Fig. 6:**
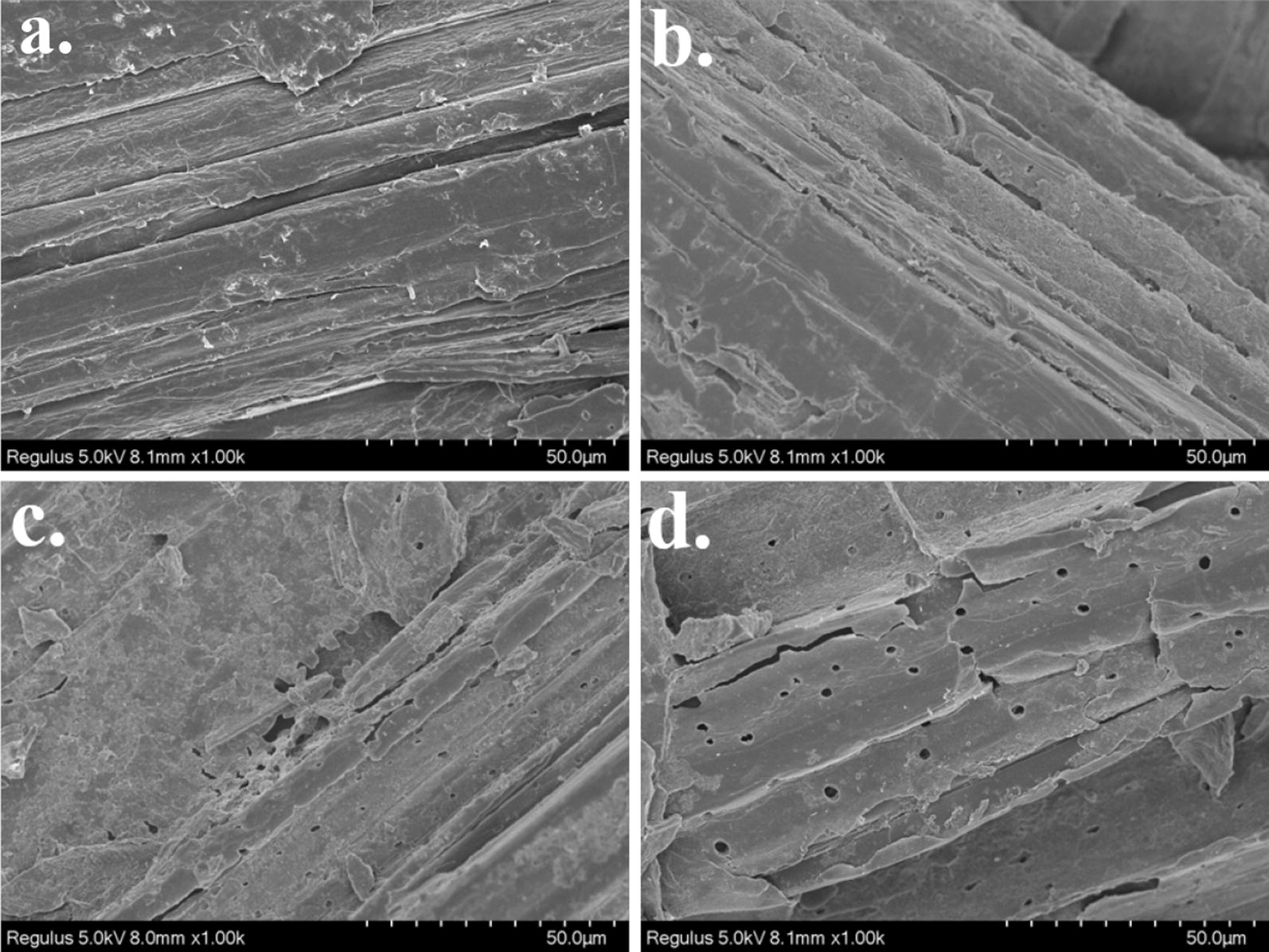
External surface of alkali-pretreated elephant grass by SEM imaging. **a** The elephant grass before hydrolysis, **b** the elephant grass after hydrolysis. **c** The elephant grass hydrolyzed with addition of BSA, and **d** with addition of Trx-His-S

Figure 6 shows the SEM imaging of the residual surficial structure of elephant grass after hydrolysis. After hydrolysis, with the addition of BSA and Trx-His-S, many holes were observed on the surficial structure of lignocellulose. The holes on the lignocellulose surface led to the increased accessibly of cellulose to cellulase and an improvement in enzymatic hydrolysis yield [30]. Addition of Trx-His-S yielded more holes on the surface of the substrate compared with BSA, which confirmed the better influence of Trx-His-S on hydrolysis compared with BSA.

### The supposed effects of Trx-His-S properties on the hydrolysis

The purpose of a number of initial experiments was to compare the binding capability of CBMs and FnIIIs to lignin, using Trx-His-S as control [14, 15, 31]. Trx-His-S showed a higher binding affinity to lignin than CBMs and FnIIIs. After adsorption to lignin, the Trx-His-S in the supernatant remained almost undetected. Likely, addition of Trx-His-S could block the lignin binding sites and reduce the nonproductive adsorption of cellulase to lignin, which benefitted the enzymatic hydrolysis of lignocellulose. EG-AP contained high contents of lignin; therefore, addition of Trx-His-S relaxed the limit of lignin and caused visible improvement in the hydrolysis yield.

**Fig. 7:**
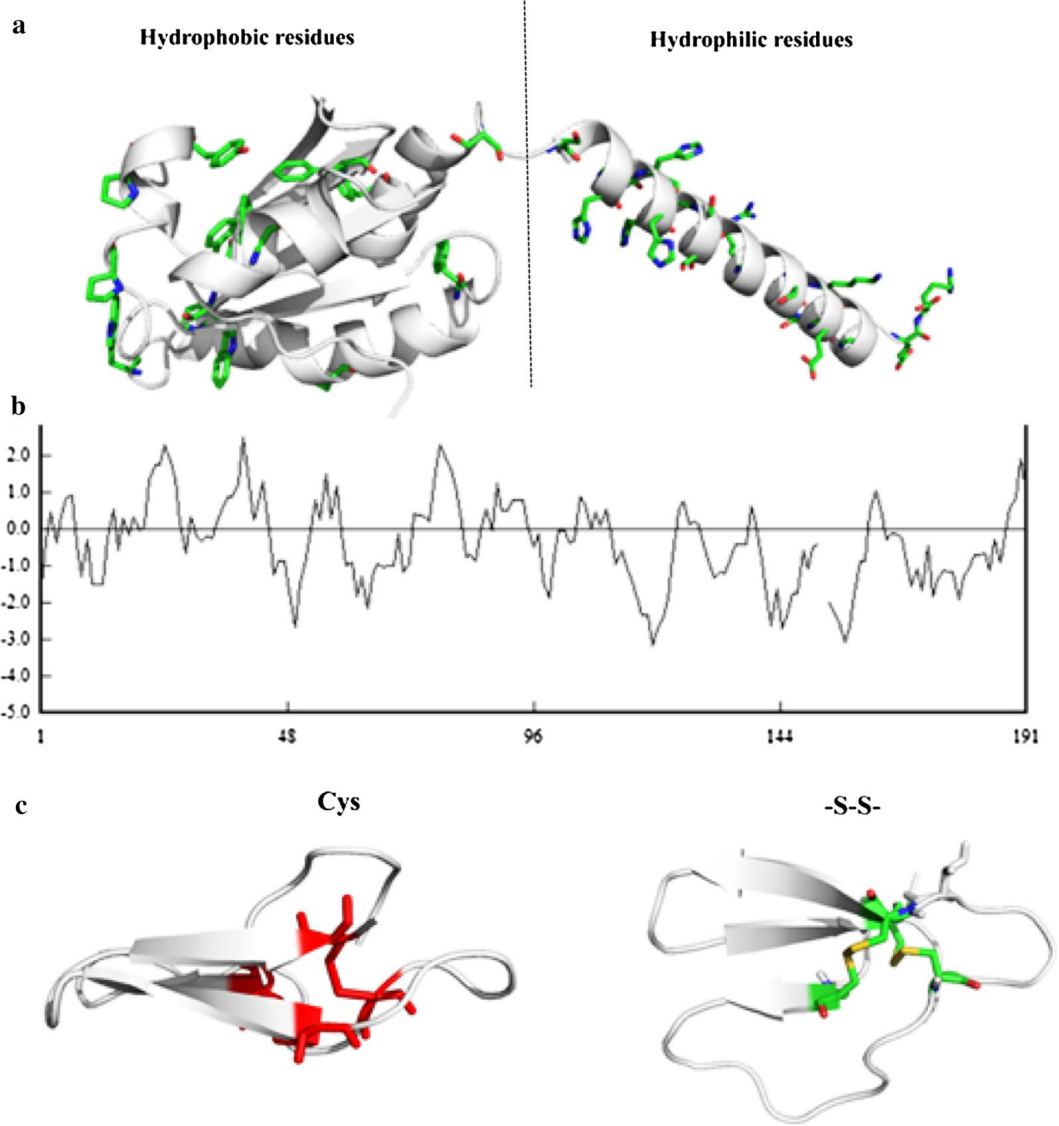
**a**Distribution of amino acids on molecular surface and **b** the hydrophobicity of amino acids in Trx-His-S. **c** Cystines and disulfide bonds (yellow) on the carbohydrate binding domains in *endo*-beta-1,4-glucanase

Trx, which was first characterized in *E. coli*, is a 12 kDa multifunctional protein with a conserved redox catalytic site (–Cys–Gly–Pro–Cys–). Trx is a type of redox-active protein that plays a vital role in maintaining the cellular environment at the reduced state [32]. pET32a is a type of Trx·Tag commercial vector, which could express the Trx protein in *E. coli* DE3. During the protein expression process in *E. coli* DE3, Trx has the capability to catalyze the form of disulfide oxide in protein, promote the dissolution of proteins, and prevent the formation of inclusion bodies [33, 34]. It has also been reported that Trx could protect the protein from being digested by proteases. Thus, addition of Trx may also protect cellulase from being digested by protease and stabilize the cellulase during the hydrolysis process. Analyzing the filter paper activity (FPA) of cellulase secreted by *Penicillium oxalicum* showed that Trx could increase the FPA of cellulase from 0.19 FPU/mL to 0.21 FPU/mL under hydrolytic conditions. Furthermore, two disulfide bonds existed on the binding surface of the carbohydrate binding domain in *endo*-beta-1,4-glucanases. The carbohydrate binding domain played an important role in the binding of *endo*-beta-1,4-glucanases to cellulose, as shown in Fig. 7. The existence of Trx during hydrolysis may stabilize the disulfide bonds in the carbohydrate binding domain and promote the hydrolysis process.

Analyzing the amino acid sequence and molecular surface properties demonstrated that the Trx-His-S had a high hydrophobic solvent-accessible surface area on the head and a long hydrophilic linker, as shown in Fig. 7. Hydrophobicity has been reported to be the main driving force for cellulase binding to lignin [29, 35]. Consequently, the exposed hydrophobic patches of Trx-His-S could compete with the cellulase to interact with lignin [12, 29]. This may be the reason for the high adsorption affinity of Trx-His-S to lignin, which was beneficial for the hydrolysis process. However, further studies are required to identify the mechanism of its role in enzymatic hydrolysis.

## Conclusions

Elephant grass and reed straw could feasibly be used as feedstocks for bioconversion. However, their hydrolysis performance was limited by lignin, which was identified as droplets on the elephant grass surface and as high content in reed straw. Organosolv and alkali pretreatments improved enzymatic sugar production; however, the increase in hydrolysis yield of pretreated elephant grass was not as effective as that of reed straw. Addition of BSA and Trx-His-S visibly improved the hydrolysis yield of alkali-pretreated elephant grass. Particularly, Trx-His-S performed well as an additive during bioconversion, and its structural and catalytic capabilities were beneficial to enzymatic hydrolysis.

## Materials and methods

### Materials

Corn stover and wheat straw were collected from Liaocheng, Shandong Province, China. Reed straw was collected from Jinan, Shandong Province, China. Elephant grass was collected from Danzhou, Hainan Province, China. Enzymatic residual lignin (EHL) was isolated as described before [36]. Commercial cellulase was purchased from Sino Biotechnology Co., Ltd. in solid powder form with filter paper activity of 160 FPU/g [37]. BSA was purchased from Sigma-Aldrich, St. Louis, MO, USA.

The Trx-His-S protein was expressed in *E. coli* by transferring the vacant vector pET 32a (which carries the Trx encoding gene) into *E. coli* DE3. The protein Trx-His-S was produced as described in the pET System Manual.

### Analytic methods

The chemical components of substrate were detected following the methods of the National Renewable Energy Laboratory (USA). In brief, anhydrous ethanol was firstly used to extract the straws. The residues were dried at room temperature and were completely hydrolyzed by sulfuric acid to monosaccharides. The monosaccharides in the filtered acid-hydrolyzed liquid were measured by high-performance liquid chromatography (HPLC, Shimadzu, Kyoto, Japan), and the contents of cellulose and hemicellulose were calculated as reported before [36]. Acid-hydrolyzed residues were collected and dried off at 105 °C, which were then dried to ash at 575 °C. The lignin content was calculated by mass difference before and after dry ashing treatment.

The substrate was dried at 50 °C for 24 h and then used to collect the imaging and energy dispersion spectrum (EDS) analyses by a Regulus8220 scanning electron microscope (SEM, Hitachi, Japan). The SEM images were collected with a magnification of 1.00 k at 5.0 kV. The EDS was measured with a magnification of 1000 at 10.0 keV.

The X-ray diffraction (XRD) patterns of the feedstock were measured by Bruker AXS D8 ADVANCE which was equipped with a Lynxeye 1-D detector (Bruker AXS GmbH, Karlsruhe, Germany). A diffractometer with Cu Kα radiation was used to generate the XRD data at 40 kV and 40 mA with an angular range of 2*θ* = 5°–35°. The divergence angle was 0.2°, the step size was 0.02°, and the acquisition time was 2 s per step.

### Pretreatments

Organosolv and alkali pretreatments were conducted to change the lignocellulose characteristics. The ethanol organosolv pretreatment was performed with 60% ethanol containing 1.25% sulfuric acid, at 180 °C for 20 min [7]. The alkali pretreatment was conducted with 0.5 M NaOH at 80 °C for 1 h. After pretreatment, the pH value of the slurry was adjusted to pH 3.0 by sulfuric acid. In the above two pretreatments, the ratio of solid to liquid was 10:1. After pretreatment, the residual solid was filtered and washed by tap water until pH became neutral. The residuals were stored in ziplock bags and stored at 4 °C for subsequent experiments.

### Hydrolysis experiments

The hydrolysis experiments were performed in 50 mM acetate buffer (pH 4.8), with 10% solid substrate consistency and incubated at 50 °C under 150 rpm for 72 h. The commercial cellulase loading was 15 FPU/g dry solid substrate. BSA and Trx-His-S were added to the hydrolysis with a dosage of 2 mg protein/g solid substrate. After hydrolysis, the supernatant was collected by centrifugation and was filtered through a 0.22-μm Micron PES filter. The reducing sugars in the supernatant were measured by HPLC (Shimadzu, Kyoto, Japan) with Shimadzu LC-10AD detector. The HPLC was performed in a Bio-Rad HPX-87H column with 10 μL injected volume at 60 °C with 5 mM H_2_SO_4_ as eluent at a flow rate of 0.4 mL/min.

### Adsorption of Trx-His-S to EHL

EHL and Trx-His-S were mixed together in a 1 mL reaction system in 50 mM acetate buffer. The protein concentration was increased to the solubility of Trx-His-S in the reaction buffer, and the dosage of lignin was 0.03 g/mL as described before [38]. After adsorption, the protein in the supernatant was centrifuged and analyzed by sodium dodecyl sulfate polyacrylamide gel electrophoresis (SDS-PAGE) in a 12% polyacrylamide gel (Bio-Rad) [36].

## Data Availability

Not applicable.

## Abbreviations

SDS-PAGE: sodium dodecyl sulfate polyacrylamide gel electrophoresis
BSA: bovine serum albumin
Trx-His-S: thioredoxin with His- and S-Tags
CBMs: carbohydrate-binding modules of cellulases
FnIIIs: fibronectin III-like domains
CS: corn stover
WS: wheat straw
RS: reed straw
EG: elephant grass
EDS: energy dispersion spectrum
XRD: X-ray diffraction
EHL: enzymatic residual lignin
